# Acquired Gerbode Defect in a Patient With Infective Endocarditis of Bicuspid Aortic Valve

**DOI:** 10.7759/cureus.15352

**Published:** 2021-05-31

**Authors:** Mohammed Mahdi, Muhammet Ozer, Bipinpreet Nagra, Patrick Aufiero, Bharat Kantharia

**Affiliations:** 1 Internal Medicine, Capital Health Regional Medical Center, Trenton, USA; 2 Cardiovascular Disease, Capital Health System, Trenton, USA; 3 Infectious Diseases, Capital Health System, Trenton, USA; 4 Cardiovascular Disease, Mount Sinai Hospital, New York, USA

**Keywords:** acquired, gerbode defect, infective endocarditis, streptococcus agalactiae, bicuspid aortic valve

## Abstract

Aortic valve abscess is a fatal complication of infective endocarditis. Transthoracic echocardiography is the initial imaging obtained in suspected infective endocarditis. However, its accuracy in detecting cardiac complications remains low, thus should be followed by transesophageal echocardiography if the clinical situation permits. Here, we present a case of a bicuspid aortic valve infective endocarditis caused by S*treptococcus agalactiae* and complicated with aortic valve abscess and acquired Gerbode defect, which appeared as a tricuspid valve vegetation on transthoracic echocardiography.

## Introduction

Infective endocarditis (IE) is a potentially life-threatening infection of the cardiac endothelial layer. It is considered to be rare with incidence of 11 per 100,000 people in the United States [1]. Systemic embolization, heart failure, and perivalvular abscess are common complications associated with increased mortality approaching 25% worldwide [2]. Gerbode defect is a ventricular septal defect (VSD) between the left ventricle and the right atrium, named after the cardiothoracic surgeon Frank Gerbode, described in five surgically repaired cases in 1958 [3]. Gerbode defect is mainly a congenital defect, however, acquired defects which could be iatrogenic due to surgical intervention and non-iatrogenic due to infective endocarditis or inferior wall myocardial infarction have been described [4]. We present a case of acquired Gerbode defect due to infective endocarditis of bicuspid aortic valve caused by *Streptococcus agalactiae*.

## Case presentation

An 18-year-old male presented to the emergency department complaining of fever, shortness of breath, and productive cough associated with nausea, headache, and chills of five days duration. His symptoms did not improve with outpatient treatment of azithromycin antibiotic and albuterol inhaler. He was living in a college dormitory and denied smoking or recreational drug use. He had no history of known congenital heart disease. Prior to his current illness, he had been very healthy except for a brief illness with Epstein-Barr virus mononucleosis in his childhood. He had no physical limitations and regularly engaged in sports activities in his college. He had a wisdom tooth extraction performed four months prior to the current presentation. On presentation blood pressure was 118/40 mmHg, heart rate 94 beats per minute, respiratory rate of 20 per minute, temperature 102 degrees Fahrenheit orally, and oxygen saturation of 97% on ambient air. Physical examination was remarkable for loud harsh pansystolic murmur with thrill and a soft diastolic murmur. Lung auscultation revealed bibasilar crackles. Neck veins were non-distended, and lower extremity edema was not detected. 

Investigations

**Figure 1:**
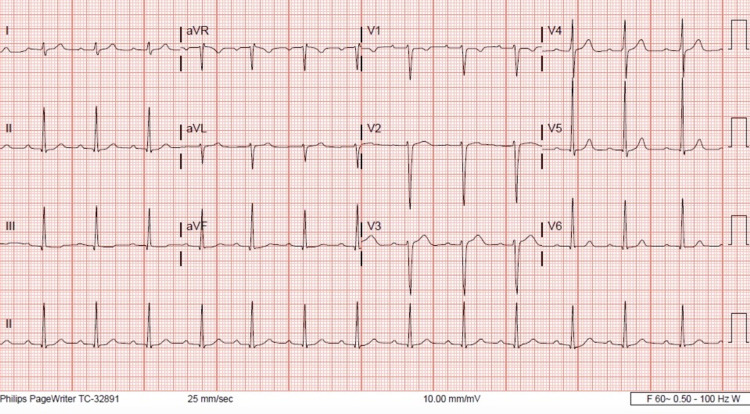
12-lead electrocardiogram Normal sinus rhythm with a prolonged PR interval of 220 msec, consistent with first-degree atrioventricular block.

Initial laboratory test results were remarkable for white blood cell count of 10.3x10^3^/ul (4-10x10^3^/ul), platelets of 95x10^3^/ul (150-400x10^3^/ul), and procalcitonin of 7 ng/ml (0-0.5 ng/ml). Renal and liver function tests were within normal limits. Two sets of blood cultures were positive for gram positive cocci in pairs and chains initially, which were identified as S*treptococcus agalactiae* (group B *Streptococcus*) 48 hours later. Urine analysis was negative for *Streptococcus pneumonia* and *Legionella pneumophila* antigens. Chest X-ray was evident for left lower lobe opacity concerning for pneumonia. Urine culture, CT scan of the head, and toxicology screen in urine were unremarkable. Electrocardiogram showed normal sinus rhythm, and a first-degree heart block with a PR interval of 221 msec (Figure 1).

**Figure 2:**
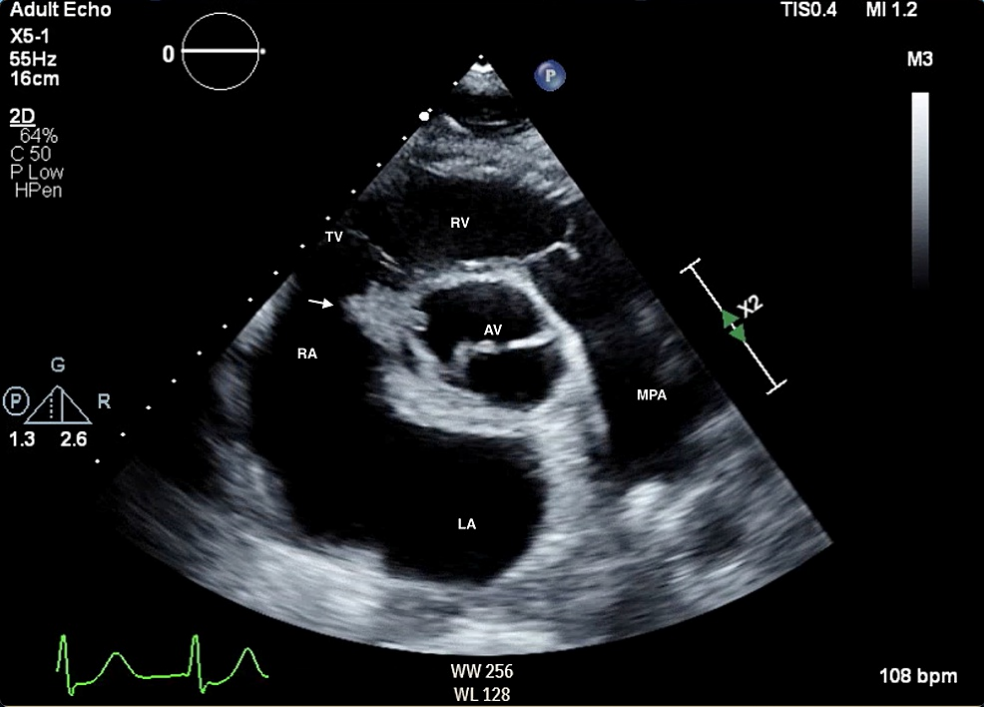
Transthoracic echocardiogram, parasternal short-axis view Demonstrates bicuspid aortic valve with a complex vegetation involving the aortic root and extending into the right atrium abutting the tricuspid valve (arrow). RV: right ventricle, TV: tricuspid valve, RA: right atrium, AV: aortic valve, LA: left atrium, MPA: main pulmonary artery.

**Figure 3:**
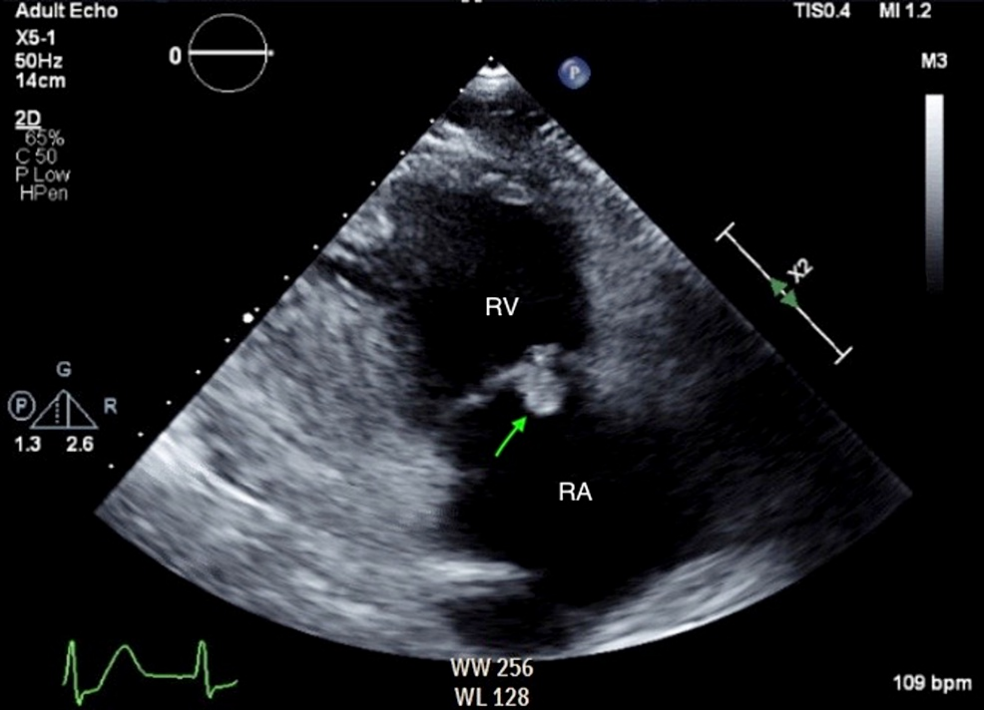
Transthoracic echocardiogram, right ventricle inflow view Shows a right atrial vegetation or mass (arrow) that looks like tricuspid valve vegetation. RA: right atrium, RV: right ventricle.

A transthoracic echocardiogram (TTE) showed aortic valve with bicuspid morphology, aortic valve vegetation, and an echo-dense mass in the right atrium that seemed to be tricuspid valve vegetation (Figures 2, 3; Videos 1, 2).

**Video 1:** Transthoracic echocardiogram, right ventricular inflow view Shows right atrial vegetation that can be mistaken as tricuspid valve vegetation.

**Video 2:** Transthoracic echocardiogram, parasternal short-axis view Shows bicuspid aortic valve with aortic valve vegetation at the right coronary cusp.

The left ventricle was moderately dilated with an estimated left ventricular (LV) ejection fraction of 60%. Also, there was moderate tricuspid regurgitation, moderate pulmonary hypertension, aortic insufficiency, and small pericardial effusion (Videos 3, 4).

**Video 3:** Transthoracic echocardiogram, parasternal long-axis view Shows moderately dilated left ventricle, deformed/thickened aortic valve, and minimal pericardial effusion.

**Video 4:** Transthoracic echocardiogram, parasternal long-axis view with color doppler Shows aortic valve regurgitation.

**Figure 4:**
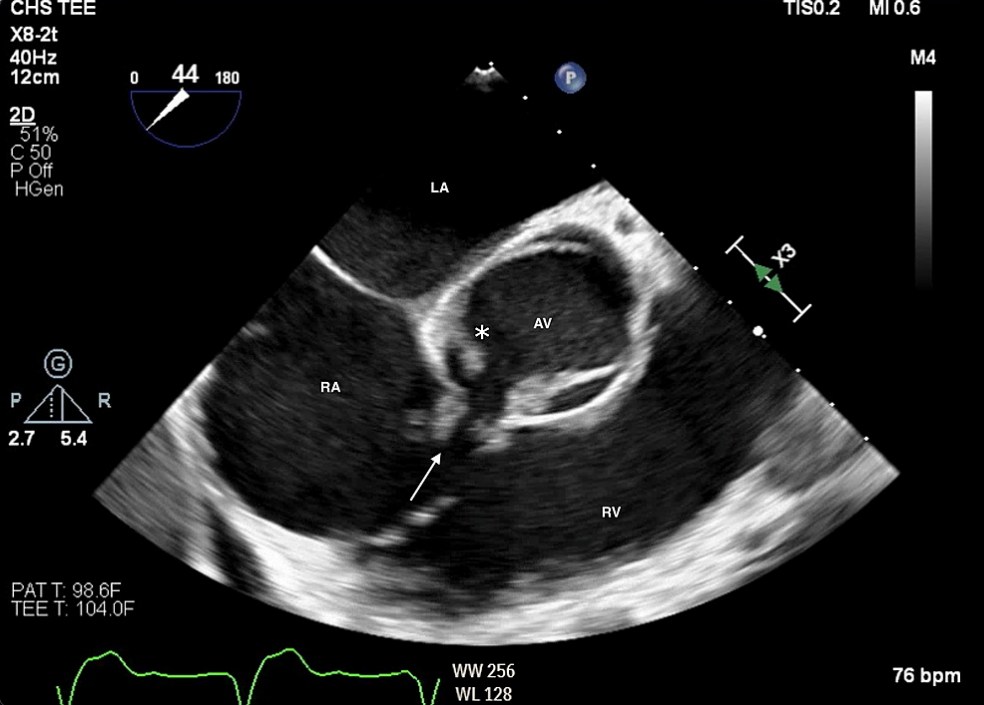
Trans-esophageal echocardiography, mid-esophageal short-axis view Demonstrates a bicuspid aortic valve (AV) with perforated leaflet (asterisk) and ruptured aortic root into right atrium (RA) forming Gerbode defect (arrow). LA: left atrium, RV: right ventricle.

Subsequently, a trans-esophageal echocardiography (TEE) was performed, which confirmed aortic valve of bicuspid nature, a large aortic valve vegetation, and aortic valve abscess with left ventricular outflow tract defect to right atrium (LVOT-RA) consistent with Gerbode defect (Figure 4; Videos 5, 6, 7). Additionally, aortic valve cusp/leaflet perforation was noticed (Videos 8, 9).

**Video 5:** Trans-esophageal echocardiography, mid-esophageal short-axis view Shows aortic valve abscess with aortic root rupture into the right atrium close to the tricuspid valve forming Gerbode defect.

**Video 6:** Trans-esophageal echocardiography, mid-esophageal short-axis view Put close view to the aortic valve abscess demonstrating the typical hypodense areas.

**Video 7:** Trans-esophageal echocardiography, mid-esophageal short-axis view with color doppler Demonstrates the flow through Gerbode defect.

**Video 8:** Trans-esophageal echocardiography, mid-esophageal short-axis view Shows ruptured/flail leaflet of bicuspid aortic valve.

**Video 9:** Trans-esophageal echocardiography, mid-esophageal long-axis view Shows fenestrated anterior leaflet of bicuspid aortic valve.

Management

Initially, the patient was treated empirically for infective endocarditis with intravenous vancomycin and ceftriaxone. While undergoing TEE, the patient developed cardiac arrest with pulseless electrical activity (PEA). Return of spontaneous circulation was achieved after 18 minutes of cardiac resuscitation. The patient was promptly transferred to a tertiary cardiac center for an emergent surgical intervention. Intra-operatively, bicuspid aortic valve, ruptured aortic valve abscess, ventricular septal defect (VSD), and left ventricular outflow tract to right atrial defect (LVOT-RA) were identified. He underwent surgical composite mechanical aortic root replacement, debridement and patch to LVOT, VSD closure, and right ventricular epicardial bipolar leads placement. He made successful recovery post-operatively with inpatient rehabilitation and continuation of intravenous antibiotic therapy. 

Follow-up

Two months later, the patient developed acute pulmonary edema due to a dehiscent prosthetic aortic valve. He underwent another surgical aortic root replacement. On an outpatient follow up visit, the patient has a stable functioning graft with preserved ejection fraction and asymptomatic.

## Discussion

*Staphylococcus aureus* is the most common microorganism causing IE in North America (43%) followed by coagulase-negative S*taphylococci* (12%) [5]. *Streptococcus agalactiae* is an unusual organism implicated in native valve endocarditis with frequent complications, including thromboembolic event, heart failure, and cardiogenic shock. The mortality rate of S*treptococcus agalactiae*-induced IE is up to 50% in a case series [6]. Although bacteremia without focus is the most common presentation of invasive *Streptococcus agalactiae* disease in non-pregnant adults, endocarditis is not rare and constitutes about 10% of invasive *Streptococcus agalactiae* disease [7]. *Streptococcus agalactiae* is known to colonize the genitourinary tract, however, oral cavity through oral sexual intercourse and food borne transmission has been reported [8]. In this case, the time needed for left ventricle to be moderately dilated suggests that the source of bacteremia is likely to be dental procedure than pneumonia.

A negative result of TTE is not sufficient to rule out IE if the clinical suspicion is present. Also, TEE has better sensitivity and specificity in evaluating intra-cardiac complications such as abscess and should be considered when clinical situation allows [9]. An intracardiac abscess is one of the most dismal complications of IE with high mortality and morbidity rates that usually predilects the aortic valve [10]. Perivalvular abscess formation tends to corrupt conductive system and cause an atrioventricular (AV) block [11]. In this case, the TTE was remarkable for aortic valve vegetation and right atrial oscillating mass. Although, it seemed like a tricuspid valve vegetation on the first look, its separation from the tricuspid valve cusps raised the concerns of ruptured aortic abscess, especially given the aortic insufficiency and AV block. TEE not only clearly identified ruptured aortic valve abscess, but it further revealed a LVOT-RA (Gerbode) defect.

The proposed pathophysiological mechanisms of Gerbode defect usually are reopening of a congenital defect, widening of small defect, or destruction of the membranous ventricular septum [12]. In our opinion, the Gerbode defect observed in our patient is an acquired defect rather than congenital given the subvalvular location and close proximity to the ruptured aortic valve abscess. The current approach in the management of acquired Gerbode defect secondary to IE is surgical repair of the defect as transcatheter device is not an option due to the presence of infection [13].

It is to be noted that the patient had poor dentition and tooth extraction procedure, which would raise the question whether pre-operative antibiotic prophylaxis could have prevented this drastic complication. The current American Heart Association/American College of Cardiology (AHA/ACC) guidelines do not include a bicuspid aortic valve in a high-risk group category for pre-operative IE antibiotic prophylaxis [14]. However, in a recent study, the bicuspid aortic valve was found to carry a substantially increased risk of IE and intracardiac complications [15].

## Conclusions

Infective endocarditis due to *Streptococcus agalactiae* carries poor prognosis, and in this case, was associated with aortic valve abscess and Gerbode defect. Atrioventricular block in any degree should raise the suspicion of aortic valve abscess and trans-esophageal echocardiogram should be considered as early as possible to prevent misdiagnosis and thence grave outcomes. Infective endocarditis prophylaxis guidelines may be revisited in future to include antibiotic prophylaxis patients with bicuspid aortic valve.
